# Supplementary material for: Comparative genomics provides new insights into the diversity, physiology, and sexuality of the only industrially exploited tremellomycete: Phaffia rhodozyma
Source: BMC Genomics. 2016 Nov 9;17:901. doi: 10.1186/s12864-016-3244-7 (PMC5103461; doi:10.1186/s12864-016-3244-7)
Supplement: Additional file 6: — List of orphan genes with links to PFAM (related to Additional file 1: Table S1). (ZIP 1428 kb) [file 12864_2016_3244_MOESM6_ESM.zip › PFAM_UNIQUE/G00401_P.html]

#                                                                            --- full sequence --- -------------- this domain -------------   hmm coord   ali coord   env coord
  
# target name        accession   tlen query name           accession   qlen   E-value  score  bias   #  of  c-Evalue  i-Evalue  score  bias  from    to  from    to  from    to  acc description of target
  
#------------------- ---------- ----- -------------------- ---------- ----- --------- ------ ----- --- --- --------- --------- ------ ----- ----- ----- ----- ----- ----- ----- ---- ---------------------
  
G00401\_P             -            197 PSRT                 PF07636.6     32       8.1    4.7   7.8   1   2     0.013        38    2.6   0.2     6    13    33    40    30    55 0.86 - PSRT  
G00401\_P             -            197 PSRT                 PF07636.6     32       8.1    4.7   7.8   2   2    0.0053        16    3.8   2.8     7    16   164   173   158   181 0.84 - PSRT  
G00401\_P             -            197 Rab15\_effector       PF15208.1    236     0.026   12.6   0.0   1   1   1.2e-05     0.037   12.1   0.0   173   231    21    80     7    83 0.77 - Rab15 effector
